# Range Extension of the Popeye Catalufa (*Pristigenys serrula*, Gilbert 1891) to Central Chile During the “El Niño” Southern Oscillation (ENSO) 2023–2024

**DOI:** 10.1002/ece3.70720

**Published:** 2024-12-16

**Authors:** Cynthia M. Asorey, Jeremías Cuevas, María Angélica Larraín, Cristian Araneda

**Affiliations:** ^1^ Centro de Ecología y Manejo Sustentable de Islas Oceánicas (ESMOI), Facultad de Ciencias del Mar Universidad Católica del Norte Coquimbo Chile; ^2^ Sala de Colecciones Biológicas, Facultad de Ciencias del Mar Universidad Católica del Norte Coquimbo Chile; ^3^ S.T.I. Pescadores Caleta Zapallar Francisco de Paula S/N Valparaíso Chile; ^4^ Food Quality Research Center Universidad de Chile Santiago Chile; ^5^ Departamento de Ciencia de los Alimentos y Tecnología Química. Facultad de Ciencias Químicas y Farmacéuticas Universidad de Chile Santiago Chile; ^6^ Departamento de Producción Animal. Facultad de Ciencias Agronómicas Universidad de Chile Santiago Chile

**Keywords:** Bigeyes, epipelagic fishes, Pricanthidae, septentrional invaders, southeast Pacific

## Abstract

We report the presence of *Pristigenys serrula* Gilbert 1891 off Zapallar (−32.568253, −71.464225), central Chile, during the ENSO 2023–2024 event. Morphology and a partial fragment of the mitochondrial markers cytochrome oxidase 1 (*COX*1) and *16S rDNA* identified one specimen fished by artisanal fishermen. This report extends the known southern range limit of *P. serrula* by 9° of latitude (~1000 km) to central Chile (−32°) during strong negative ENSO conditions.

## Graphical abstract

Phylogenetic and morphological analyses confirm *P. serrula* in central Chile (32° S). The geographical distribution of *P. serrula* in central Chile extends 9° southward. This extension is associated with the “El Niño” Southern Oscillation (ENSO).

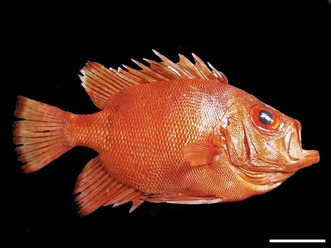

## 1 Introduction

**FIGURE 1:**
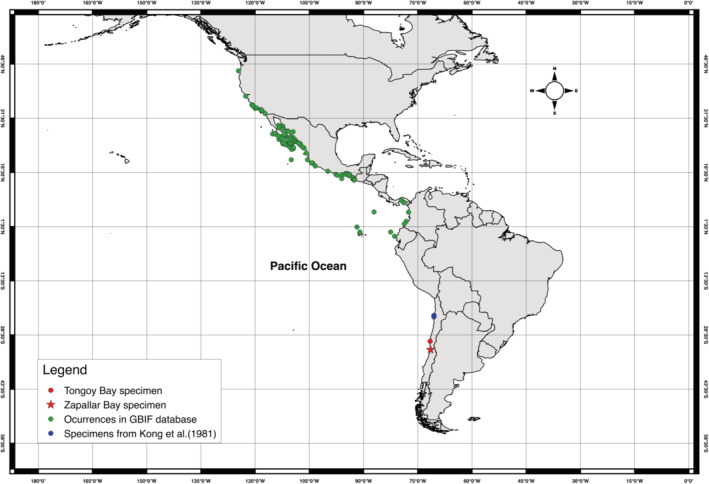
Map of distribution of *Pristigenys serrula* in the Pacific Ocean. Green dots occurrences downloaded from GBIF.org. Blue dot specimens were reported by Kong, Tomicic, and Guerra (1981). Red dot specimen collected in Tongoy Bay, Chile (August 2023). Red star specimen was collected in Zapallar Bay, Chile (February 2024) and reported in this work.

Pricanthidae fishes, called Bigeyes or catalufas because of their large eyes with a reflective layer, are carnivorous with nocturnal habits distributed worldwide in tropical and subtropical oceans (Nelson, Grande, and Wilson 2016). There are 22 described species distributed in four genera: *Cookeolus*, *Heteropriacanthus*, *Priacanthus*, and *Pristigenys* (Fricke, Eschmeyer, and van der Laan 2024). *Pristigenys serrula* Gilbert (1891) is commonly distributed in the Eastern Pacific, from Monterey Bay in California, USA, along the continental shelf to Peru, including Revillagigedo, Cocos, and the Galapagos Islands (Starnes 1988; Watson 1996) (Figure 1). This species is a nocturnal fish known to occur at depths of < 5 to over 100 m in rocky habitats (Starnes 1988). Notably, this species increases its northern and southern geographic limits in the “El Niño” Southern Oscillation (ENSO) years (Kong, Tomicic, and Guerra 1981; Starnes 1988). GBIF data indicate the more northern and southern occurrences in Oregon, US (46.18333, −123.98333, 1992) and Coquimbo, Chile (−30.22039, −71.57237, 2023) (GBIF 2024), precisely in ENSO years.

The Humboldt Current System influences the biota of the coast of Chile and southern Peru (Thiel et al. 2007). The effect of the southeast Pacific subtropical anticyclone dominates the wind force, creating equatorward, upwelling‐favorable winds along most South American Pacific coasts with latitudinally varying seasonality (Thiel et al. 2007). Weakening of upwelling intensity and cyclic ENSO events of variable intensity affect species considerably (e.g., Arntz et al. 1987; Carstensen et al. 2010; Sielfeld et al. 2010; Valqui et al. 2021; Vásquez and Vega 2004). In northern Chile around 100 species of fish normally found at lower latitudes have been reported during ENSO events. These are called “Septentrional invaders” (Sielfeld et al. 2010). Most of them have epipelagic habits (> 50%) (Sielfeld et al. 2010).

*Pristigenys serrula* was the first found off Antofagasta (23° S) on the Chilean coast during ENSO 1963–1964 (Alberti 1963). Between December 1978 and February 1979, Kong, Tomicic, and Guerra (1981) reported the presence of four specimens from San Jorge Bay, Antofagasta. The last reports of the presence of *P. serrula* in waters off Antofagasta date from 1993 to 1998–1999, probably associated with ENSO 1992–1993 and 1997–1998. In August 2023, another specimen of *P. serrula* was caught in Tongoy Bay (30° S), gbif ID 4453947964 (GBIF 2024). Until the publication of this manuscript, this specimen was alive in the aquarium of Facultad de Ciencias del Mar, Universidad Católica del Norte, Chile (Jeimy Aguirre, Aquarium Manager, pers. comm.).

## 2 Materials and Methods

### 2.1 Sample and Taxonomic Identification

**FIGURE 2:**
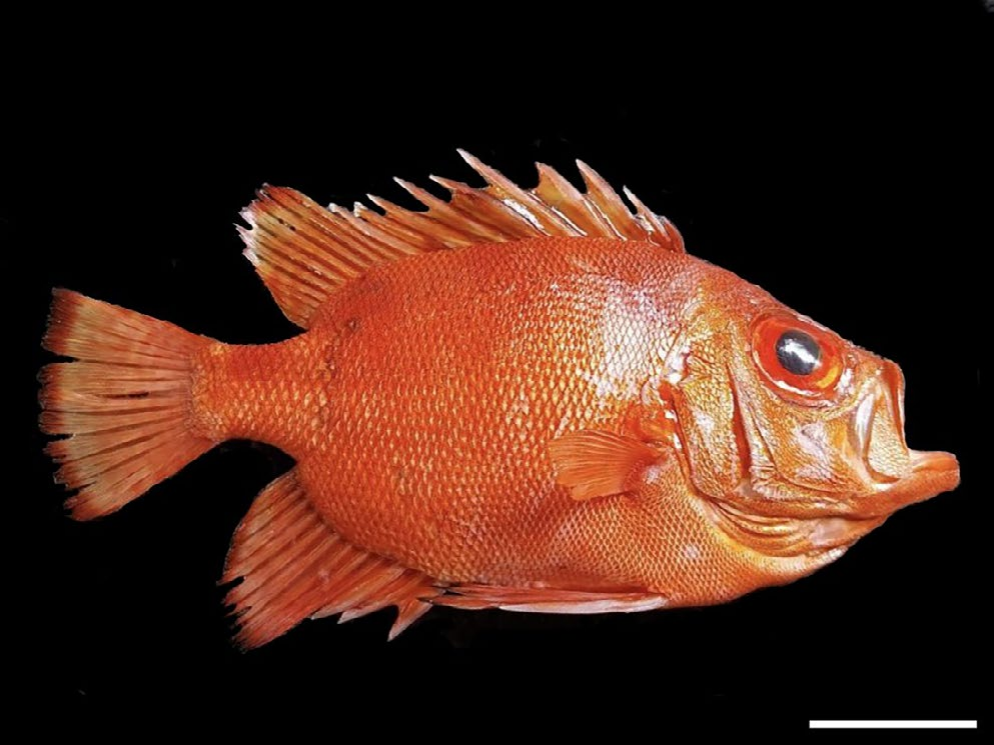
Picture of *Pristigenys serrula* specimen caught in Zapallar Bay (Chile) showing the external diagnostic traits. Scale bar: 5.0 cm (photo credit: Claudio Cisternas).

On February 09, 2024, an experienced artisanal fisherman captured a Popeye catalufa off Zapallar Bay (−32.568253, −71.464225), central Chile (Figure 2). A three‐layer monofilament nylon fishing net was used for the capture, placed between 40 and 60 m deep. The artisanal fisherman, well acquainted with the Chilean coastal species, immediately discerned the distinct nature of this specimen and contacted C. Araneda. A confirmation photo was promptly sent after the catch (Figure S1). The captured unsexed specimen, weighing 450 g with a total length of 24 cm and a standard length of 19.5 cm, was kept frozen by the fisherman, and later, a fin clip was obtained to perform a molecular identification at the facilities of the Food Quality Research Center (University of Chile). The specimen is red with the typical morphology of the *Prystigenis* genus: Deep and compressed body, eyes very large, oblique mouth, and lateral line curved (FAO 1984) and is deposited at the Biological Collection Room (“Sala de Colecciones Biológicas”) of the Universidad Católica del Norte, Chile (Voucher SCBUCN‐10459).

### 2.2 Morphological Identification

Morphological and meristic traits were compared following the original description of Gilbert (1891); Alberti (1963), and the specimens found in Bahia San Jorge, Antofagasta (Kong, Tomicic, and Guerra 1981). Measurements were made with a metric tape (Standard and Total Body Length) and with a Vernier caliper (0.01 mm). Scales in a lateral series and the number of spines and rays in the fins were counted with the naked eye.

### 2.3 Molecular Taxonomic Identification and Phylogenetic Analysis

Molecular identification was performed by sequencing fragments from *COX*1 and *16S rDNA* mitochondrial genes. DNA was extracted with the E.Z.N.A. Tissue DNA Kit (Omega BIO‐TEK) and used directly in the PCR reaction. Amplification conditions were 1 μL of DNA, 1x PCRBIO Taq Mix Red containing MgCL_2_, dNTPs, enhancers, stabilizers, and a red dye for agarose gel electrophoresis tracking (PCR Biosystems Ltd.), 0.2 μM primers cocktail COI‐3 from Ivanova et al. (2007) or 16sar‐L/16sbr‐H from Palumbi et al. (2002) in a final volume of 50 μL. The thermal profile considered an initial denaturation step at 95°C for 3 min, 40 cycles at 94°C for 15 s, annealing at 50°C for 30 s, and 72°C for 1 min, with a final extension at 72°C for 6 min. PCR products were cleaned up using Favorgen PCR/GEL kit, verified by horizontal electrophoresis (agarose gel 1.2%), and quantified by spectrophotometer NanoDrop 2000 (Thermo Fisher). Bidirectional Sanger sequencing was performed in Macrogen Chile Spa (Santiago, Chile) using the M13F (−21) TGTAAAACGACGGCCAGT and M13R (−27) CAGGAAACAGCTATGAC universal primers (Messing 1983). Forward and reverse raw sequences were aligned, manually edited, and trimmed using Geneious Prime 2024.0.3 (https://www.geneious.com). The *COX*1 alignment was used to obtain a phylogenetic reconstruction with the PhyML 3.3.2 plugin with the following settings: substitution model = GTR, bootstrap = 1000, the proportion of invariable sites = estimated, gamma distribution parameter = estimate, optimize = Topology/length/rate (Guindon et al. 2010).

## 3 Results

Measurements of 22 morphological and meristic characters were taken and compared with the specimen found by Alberti (1963) and three specimens deposited in the National Museum of Natural History of Chile (MNHN) by Kong, Tomicic, and Guerra (1981). Only 12 characters have been compared with the specimens from Kong, Tomicic, and Guerra (1981) and 11 with the specimen described for Alberti (1963) (Table 1).

**TABLE 1:** Comparison between meristic and morphometrics traits among specimens of *Pristigenys serrula* collected in Chilean waters.

Voucher ID	This work	Alberti (1963)	Kong, Tomicic, and Guerra (1981)*		
	SCBUCN‐10459	n.a.	MNHN 6023	MNHN 6024	MNHN 6025
Date of capture	02/09/2024	07/18/1958	02/03/1979	02/03/1979	01/13/1979
BW, body weight (g)	450	820	n.a.	n.a.	n.a.
SL, standard length (cm)	19.5	25.00	18.5	19.20	20.30
TL, total length (cm)	24.00	31.50	22.50	22.70	24.60
BD, body depth (cm)	10.48	13.50	8.55	9.65	9.99
BW, body width (cm)	3.81	n.a.	n.a.	n.a.	n.a.
CPL, caudal peduncle length (cm)	4.01	3.40	n.a.	n.a.	n.a.
CPD, caudal peduncle depth (cm)	2.79	n.a.	2.20	2.43	2.68
HL, head length (cm)	8.10	10.00	7.61	6.92	7.75
HD, head depth (cm)	9.31	n.a.	n.a.	n.a.	n.a.
HW, head width (cm)	3.28	n.a.	n.a.	n.a.	n.a.
ORB, horizontal bony orbit length (cm)	3.01	3.90	3.35	3.17	3.47
IO, bony interorbital width (cm)	1.78	n.a.	1.49	1.74	1.59
SNT, snout length (cm)	1.94	n.a.	n.a.	n.a.	n.a.
JW, length of lower jaw (cm)	3.60	3.60	3.33	3.61	3.65
D1L, length of the longest dorsal spine (cm)	5.20	n.a.	n.a.	n.a.	n.a.
D2L, length of the longest soft dorsal ray (cm)	4.77	n.a.	n.a.	n.a.	n.a.
AL, length of the longest soft anal ray (cm)	4.24	n.a.	n.a.	n.a.	n.a.
P1L, pectoral fin length (cm)	3.85	n.a.	3.91	3.74	4.63
P2L, pelvic fin length (cm)	6.20	n.a.	6.10	6.53	6.93
Number of anal fin spines/rays	III/10	III/10	III/10	III/9	III/10
Number of dorsal fin spines/rays	X/11	X/11	X/11	X/11	X/11
LS, number of scales in the lateral line	48	63	n.a.	n.a.	n.a.

Abbreviation: n.a., not available.

* Specimens deposited in the “Sala de Colecciones Biológicas de la Universidad Católica del Norte,” Coquimbo, Chile.

**FIGURE 3:**
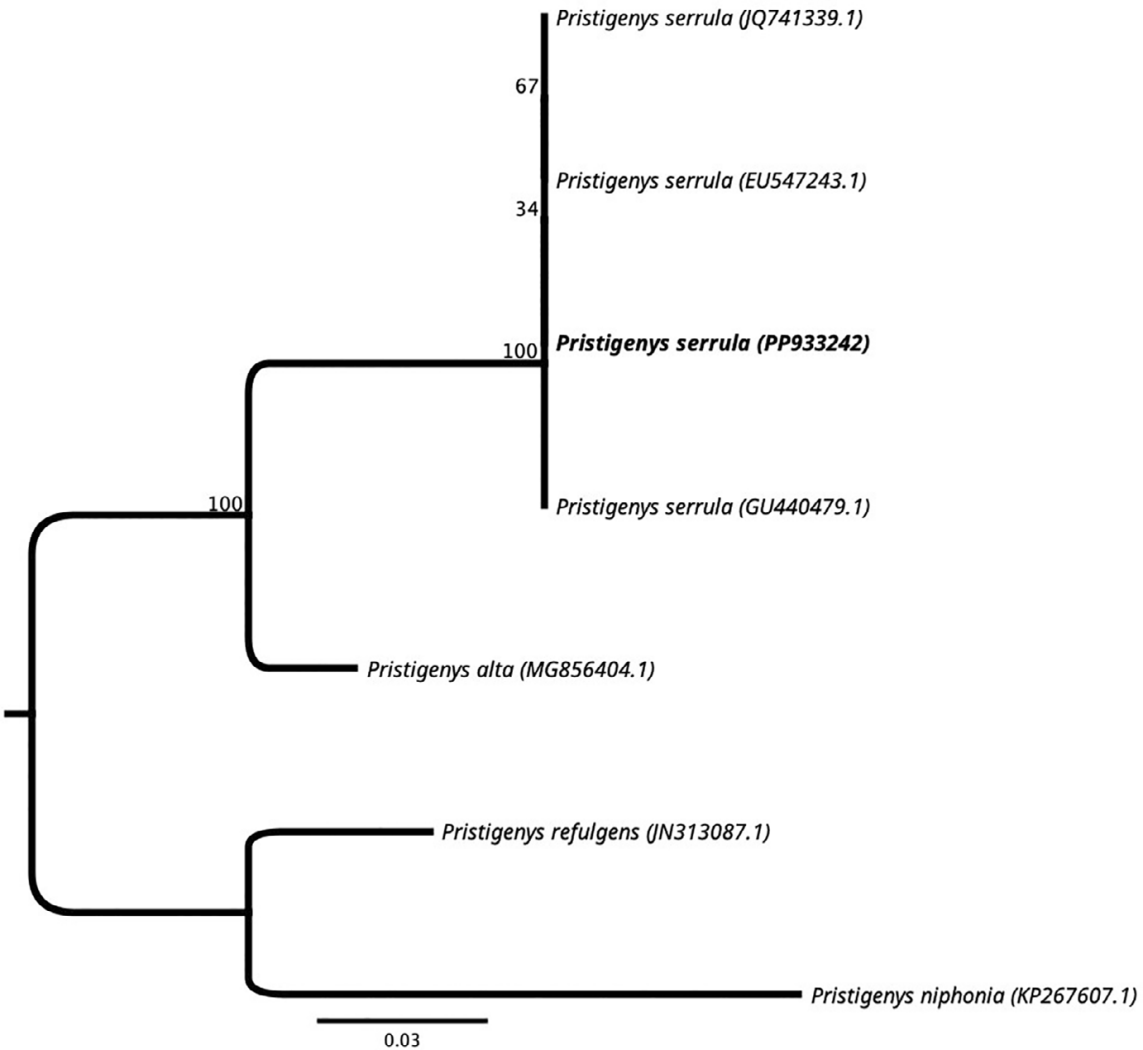
Maximum‐likelihood phylogenetic tree based on the *COX1* gene for specimens of *Pristigenys serrula* and other *Pristigenys* species mined from public databases. In bold is the specimen collected in Zapallar Bay (Chile) on February 2024 (GenBank accession PP933242).

Consensus sequences for *COX*1 and *16S rDNA* resulted in 658 bp and 615 bp, and they were uploaded to NCBI GenBank with the accession numbers PP933242 and PP933256, respectively. Four *16S rDNA* genes and six *COX*1 gene sequences of other *Pristigenys* specimens were extracted from NCBI GenBank and aligned with the ones of *P. serrula* from Zapallar. The *COX*1 sequence was identical to three *P. serrula* sequences (EU54743.1, JQ741339.1, and GU440479.1) and presented differences between 5.7% and 12.1% concerning three other *Pristigenys* species ( *P. alta* MG856404.1, *P. refulgens* JN313087.1, and *P. niphonia* KP267607.1). The *16S rDNA* sequence presented 99.4% similarity with *P. serrula* EU099463.1 and between 97.4% and 98.4% similarity with other *Pristigenys* species ( *P. alta* HQ731433.1, *P. refulgens* KF814983.1, and *P. niphonia* NC031424.1). *P. serrula* from Zapallar Bay formed a well‐defined clade with three other *P. serrula* specimens, and a high bootstrap value separates them from sequences belonging to other *Pristigenys* species (Figure 3).

## 4 Discussion

The meristic and morphometric characteristics of the *P. serrula* from Zapallar are like those reported by other authors for northern Chile, except for the number of scales in the lateral series reported by Alberti (1963) (Table 1). However, there may be an error in the count by this author because the expected range of scales in the lateral series for *P. serrula* is 45–51 (Starnes 1988). These results, combined with 100% similarity in the *COX*1 sequence and 99.4% of the *16S rDNA* sequence, confirm the presence of *P. serrula* in Zapallar Bay.

For more than 100 years, the existence of septentrional invaders has been reported in north and central Chile during ENSO conditions. The first was *Mola mola* registered by Philippi (1892) at Chañaral, Chile (−26.28, −69.86) in October 1889. Since that year, more than 100 species belonging to 86 families associated with ENSO warm events have been reported in Chile (Sielfeld et al. 2010). Some of the species are *Halichoeres dispilus* , *Masturus lanceolatus* , *Auxis rochei*, and *Scomberomorus sierra* (Sielfeld et al. 2010). However, *P. serrula* is the only species in the family Priacanthidae reported during warm conditions (Sielfeld et al. 2010). The only representative of this family in Chilean jurisdictional waters permanently is the Kara‐Kara, *Heteropriacanthus cruentatus* , on Rapa Nui Island (Easter Island) (Kong, Tomicic, and Guerra 1981).

The presence of *P. serrula* in waters off northern Chile has been widely documented during ENSO events. However, this species had not been reported further south of Antofagasta (23° S) until 2023. This report expands the recognized geographical distribution of *P. serrula* 9° (~1000 km) to the south to Chile central (32° S) during ENSO conditions. As the ENSO is one of the most noteworthy events in the Earth's climate system, the occurrence of *Pristigenys serrula* in Central Chile is only another example of how ENSO changes fish distribution. The coastal ENSO 2023–2024 has been very atypical and strong, but being recent, its effects have not yet been widely documented (Martinez‐Villalobos et al. 2024). However, mathematical models predict an increase in extreme ENSO events in the southeastern Pacific due to global climate change (Shin et al. 2022), so *P. serrula* is expected to occur again at Zapallar during warm events or even at higher latitudes.

## Author Contributions

**Cynthia M. Asorey:** conceptualization (equal), formal analysis (equal), investigation (equal), methodology (equal), writing – original draft (equal), writing – review and editing (equal). **Jeremías Cuevas:** conceptualization (equal), data curation (equal). **María Angélica Larraín:** conceptualization (equal), funding acquisition (equal), writing – review and editing (equal). **Cristian Araneda:** conceptualization (equal), investigation (equal), methodology (equal), writing – original draft (equal), writing – review and editing (equal).

## Conflicts of Interest

The authors declare no conflicts of interest.

## Supporting information


**Figure S1.** The original picture of the specimen caught in Zapallar Bay (Chile) was taken by fishermen Jeremias Cuevas, Williams Figueroa, and Claudio Cisternas.

## Data Availability

The sequences data supporting this study's findings are available in GenBank of NCBI at https://www.ncbi.nlm.nih.gov under accession numbers PP933242 (*COX1*) and PP933256 (16S rDNA).
