# Supplementary material for: Range Extension of the Popeye Catalufa (Pristigenys serrula, Gilbert 1891) to Central Chile During the “El Niño” Southern Oscillation (ENSO) 2023–2024
Source: Ecol Evol. 2024 Dec 16;14(12):e70720. doi: 10.1002/ece3.70720 (PMC11650745; doi:10.1002/ece3.70720)
Supplement: Supplementary file 1 — Figure S1. The original picture of the specimen caught in Zapallar Bay (Chile) was taken by fishermen Jeremias Cuevas, Williams Figueroa, and Claudio Cisternas. [file ECE3-14-e70720-s001.docx]

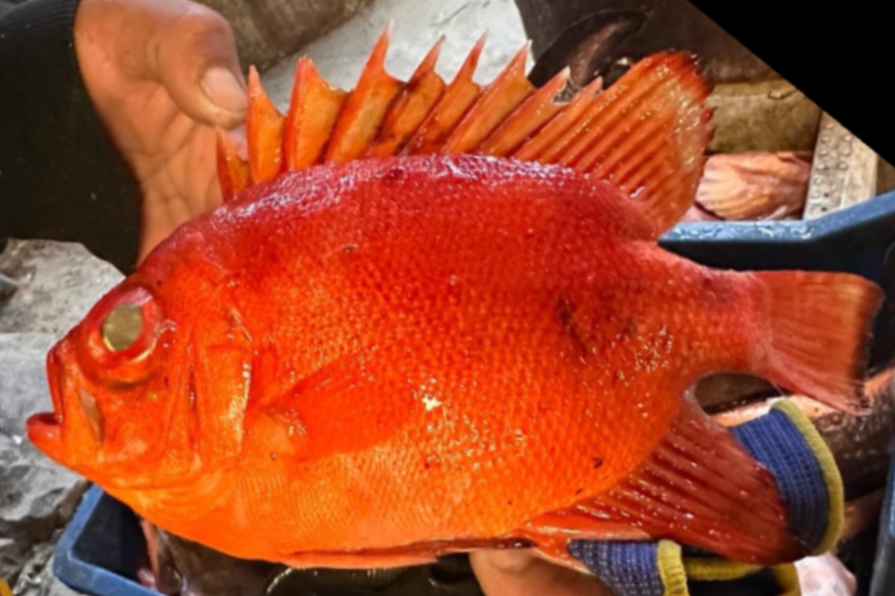


Figure S1. The original picture of the specimen caught in Zapallar Bay (Chile) was taken by fishermen Jeremias Cuevas, Williams Figueroa, and Claudio Cisternas.
